# Incorporation of Metabolic Activation in the HPTLC-SOS-Umu-C Bioassay to Detect Low Levels of Genotoxic Chemicals in Food Contact Materials

**DOI:** 10.3390/toxics10090501

**Published:** 2022-08-27

**Authors:** Emma Debon, Paul Rogeboz, Hélia Latado, Gertrud E. Morlock, Daniel Meyer, Claudine Cottet-Fontannaz, Gabriele Scholz, Benoît Schilter, Maricel Marin-Kuan

**Affiliations:** 1 Food Safety Research Department, Société des Produits Nestlé SA—Nestlé Research, Vers-chez-les-Blanc, 1000 Lausanne, Switzerland; 2 Institute of Nutritional Science, Chair of Food Science, and TransMIT Center of Effect-Directed Analysis, Justus Liebig University Giessen, 35392 Giessen, Germany

**Keywords:** hazard identification, in vitro genotoxicity, planar chromatography coupled with bioassay, food contact material, limit of biological detection, metabolic activation

## Abstract

The safety evaluation of food contact materials requires excluding mutagenicity and genotoxicity in migrates. Testing the migrates using in vitro bioassays has been proposed to address this challenge. To be fit for that purpose, bioassays must be capable of detecting very low, safety relevant concentrations of DNA-damaging substances. There is currently no bioassay compatible with such qualifications. High-performance thin-layer chromatography (HPTLC), coupled with the planar SOS Umu-C (p-Umu-C) bioassay, was suggested as a promising rapid test (~6 h) to detect the presence of low levels of mutagens/genotoxins in complex mixtures. The current study aimed at incorporating metabolic activation in this assay and testing it with a set of standard mutagens (4-nitroquinoline-*N*-oxide, aflatoxin B1, mitomycin C, benzo(a)pyrene, *N*-ethyl nitrourea, 2-nitrofluorene, 7,12-dimethylbenzanthracene, 2-aminoanthracene and methyl methanesulfonate). An effective bioactivation protocol was developed. All tested mutagens could be detected at low concentrations (0.016 to 230 ng/band, according to substances). The calculated limits of biological detection were found to be up to 1400-fold lower than those obtained with the Ames assay. These limits are lower than the values calculated to ensure a negligeable carcinogenic risk of 10^−5^. They are all compatible with the threshold of toxicological concern for chemicals with alerts for mutagenicity (150 ng/person). They cannot be achieved by any other currently available test procedures. The p-Umu-C bioassay may become instrumental in the genotoxicity testing of complex mixtures such as food packaging, foods, and environmental samples.

## 1. Introduction

The safety of complex chemical mixtures containing a substantial proportion of unidentified components is difficult to assess [1]. This is illustrated by food contact materials (FCMs) containing multiple chemicals that can potentially migrate into food and result in consumer exposure. Some of these substances are intentionally added for technological purposes. They are chemically and toxicologically well characterized. Others can be side products from manufacturing. They are referred as to non-intentionally added substances (NIAS). Among these, many may be unknown. Because not all NIAS require a thorough safety assessment, the key question is how to identify the most relevant substances on which toxicological investigations should be focused. In this context, detecting the presence of mutagenic/genotoxic/chemicals would be invaluable [2,3]. Several tests using either bacterial or mammalian cell models have been used to assess the mutagenicity/genotoxicity of FCM migrates, but in general, without establishing the suitability of the test systems used to detect sufficiently low, safety-relevant, levels of mutagens. According to previous evaluations of the suitability of mammalian cell-based reporter gene assays targeting DNA-damage endpoints, these methods lack the analytical sensitivity to fully cover genotoxic effects [4]. The bacterial reverse mutation assay, known as the Ames assay test, using *Salmonella typhimurium* or *Escherichia coli* strains as indicator organisms for direct mutagenic effects, has been considered the best option to assess FCM, even though clear limitations have been highlighted, and it cannot be used as standalone method for evaluating the genotoxic potential of FCM migrates [3,4,5,6,7]. Indeed, the assumed high potency of genotoxic/mutagenic substances requires the use of high-performance tests to detect DNA-damaging substances at very low levels. This is not achievable by any of the currently available bioassays, including the Ames test [3,4,5,6,7]. In addition, the nature of the current assay formats does not allow for the elucidation of the chemical(s) responsible for the mutagenic/genotoxic activity of a mixture, consequently complicating risk assessment. Therefore, alternative methods are needed. 

High-performance thin-layer chromatography (HPTLC) methods coupled with bioassays constitutes a very promising approach to address the limits of biological detection (LOBD). In addition, these methods provide a new, improved avenue to identify active chemicals [8,9]. HPTLC has been reported to provide an excellent separation, qualitative, and quantitative analysis of a wide range of compounds [10]. Important features of HPTLC are the application of large sample volumes and almost no solvent limitation due to evaporation before the development step and bioassay application. The chemical profiling of mixtures and the identification of bioactive bands directly from the bioautogram, with good detectability and reliability, was recently reported as eight-dimensional hyphenation [11]. The bacterial-based SOS-Umu-C bioassay was first applied on a TLC plate via a gauze-gel layer [12] and later, by spraying on the HPTLC plate [13]. In particular, the latest HPTLC coupled to SOS Umu-C (p-Umu-C) bioassay [8] has been shown as a promising solution to detect genotoxicity at low levels, e.g., the lowest effective concentration of 4-nitroquinoline-1-oxide (4-NQO) at the 20 pg/band, in complex samples, such as FCM migrates. Such a sensitive planar assay offers the potential for many further successful applications (e.g., cosmetics, new drugs, environmental and foods samples) [14]. As previously shown for environmental samples, the SOS-Umu-C test exhibits a strong concordance with the Ames test for the detection of genotoxic compounds [15,16], including mutagens.

However, the developed planar genotoxicity bioassays still suffer from important limitations. Foremost, they lack the integration of metabolic activation. Indeed, to fully assess the DNA-damage potential requires the evaluation of both parent compounds, as well as potential reactive metabolites. This addresses the fact that compounds may not be genotoxic as such and therefore, require biotransformation into reactive metabolites, for example, through cytochrome P450-mediated reactions. In in vitro test systems, metabolic activation is provided by the addition of the liver S9-fraction from rats treated with P450-inducers. Although the use of the S9-fraction has been shown to be applicable to planar assays [11,17,18], it has not been reported yet for HPTLC coupled with genotoxicity bioassays. Moreover, previously, the p-Umu-C has only been applied to a very limited number of chemicals [8]. The predictive capacity of any improved or new genotoxicity assay should be established with characterized reference substances covering different chemical classes and compared with standard tests addressing the same endpoints. Such data have not yet been reported for the p-Umu-C assay.

The main objective of this work was to incorporate the metabolic activation condition in the p-Umu-C bioassay. In addition, to address the predictive capacity of the new assay, 11 reference compounds [19] representing a broad spectrum of genotoxic mechanisms of action were tested in the presence and absence of metabolic S9-activation. To verify the potential of the p-Umu-C bioassay to detect low levels of genotoxic/mutagenic chemicals, the limits of biological detection (LOBDs) for each compound are reported and compared with those obtained in the Ames MPF test and the microtiter plate SOS Umu-C assay. Additionally, the calculated LOBDs are interpreted from the perspective of the qualifications required to document negligible carcinogenic risks. 

## 2. Materials and Methods

### 2.1. Chemicals and Materials

The bacteria strain *Salmonella typhimurium* TA1535, modified to contain the plasmid pSK1002 (PTM™ *Salmonella typhimurium* TA1535/pSK1002, cryostock), ampicillin, rat liver S9 fraction (phenobarbital/β-naphthoflavone), nicotinamide adenine dinucleotide phosphate (NADP), D-glucose-6-phosphate (G-6-P), and buffer salts solution (phosphate buffer, MgCl_2_, KCl) were obtained from Xenometrix, Allschwil, Switzerland. Ethyl acetate, methanol, and toluene (all solvents of HPLC quality), lysogeny broth (LB), D-(+)-glucose, sodium hydrogen phosphate (Na_2_HPO_4_, ≥99.0%), sodium hydroxide, potassium dihydrogen phosphate (KH_2_PO_4_, ≥99.0%), magnesium sulfate heptahydrate (MgSO_4_ · 7 H_2_O, ≥98%), potassium chloride (KCl, ≥99.0%), 4-nitroquinoline-*N*-oxide (4-NQO, CAS N° 56-57-5), aflatoxin B1 (AFB1, CAS N° 1162-65-8), mitomycin C (MMC, CAS N° 50-07-7), benzo(a)pyrene (B(a)P, CAS N° 50-32-8), *N*-ethyl nitrourea (ENU, CAS N° 759-73-9), 2-nitrofluorene (2-NF, CAS N° 607-57-8), 7,12-dimethylbenzanthracene (DMBA, CAS N° 57-97-6), 2-aminoanthracene (2-AA, CAS N° 613-13-8), methyl methanesulfonate (MMS, CAS N° 66-27-3), melamine (CAS N° 108-78-1), D-mannitol (CAS N° 69-65-8), dimethyl sulfoxide (DMSO, CAS N° 67-68-5), and fluorescein-di-β-D-galactopyranoside (FDG, CAS N° 17817-20-8) (all Sigma-Aldrich), as well as HPTLC silica gel 60 plates were purchased from Merck, Darmstadt, Germany. The plates were prewashed by elution with methanol (twin trough chamber 20 cm × 10 cm, CAMAG, Muttenz, Switzerland) up to the plate top, heated at 110 °C for 15 min (Plate Heater III, CAMAG, Muttenz, Switzerland), and stored protected from light until use. 

The Umu-C assay, including 4-NQO, 2-AA, B-buffer, stop reagent, *ortho*-nitrophenyl-β-D-galactoside (ONPG, CAS N° 369-07-3), and *Salmonella typhimurium* TA1535[psK1002], and the AMES MPF assay, including the strains TA98[pKM101, hisD3052], and TA100[pkM101, hisG46], as well as the reagents and other components, were provided by Xenometrix. The BacTiter-Glo^TM^ Microbial Cell Viability Assay (#G8230) was obtained from Promega, Dübendorf, Switzerland. 

### 2.2. Standard Solutions and Buffers 

Each stock solution was prepared at 100 mM in DMSO. Standard solutions were obtained by dilution with methanol: AFB1 at 10 and 100 pg/µL; B(a)P and MMC at 1 and 10 ng/µL; 4-NQO at 1, 10, and 100 pg/µL; ENU at 1, 10, and 100 ng/µL; MMS, D-mannitol, and melamine at 100 ng/µL; 2-NF at 0.1, 1, and 10 ng/µL; 2-AA at 10 ng/µL; and DMBA at 10 and 100 ng/µL. Phosphate buffer was prepared in purified water (H_2_O) using 40.8 g/L KH_2_PO_4_, 42.6 g/L Na_2_HPO_4_, 1.2 g/L MgSO_4_ · 7 H_2_O, and 3.7 g/L KCl, adjusted to pH 7 with solid sodium hydroxide.

### 2.3. Salmonella Typhimurium Culture

To perform the p-Umu-C assay and the liquid SOS-Umu-C assays, first, an overnight (ON) culture of the *Salmonella typhimurium* TA1535[psK1002] strain is started using 10 mL of LB medium (20 g/L LB, 1 g/L D-glucose and 50 mg/L ampicillin) inoculated with 100 µL of *Salmonella typhimurium* TA1535. The incubation is performed in a 50 mL Greiner Bio-One CellStar cell reactor tube (VWR International, Dietikon, Switzerland) for 10 h at 37 °C and 250 rpm in a shaker (Thermo Scientific digital CO_2_ resistant microplate shaker, Reinach, Switzerland). The ON incubation time of 10 h is ensured with the installation of an LED with a timer control device (ThebenHTS, theben-timer 26, Effretikon, Switzerland) in the incubator. The next day, an aliquot of the ON culture is recovered to measure the optical density (OD_600_) of the culture. The OD_600_ should be between 2.0 and 3.0 (JENWAY 6300 Spectrophotometer, Staffordshire, UK). 

### 2.4. Chromatography 

Standard solutions were applied as bands onto prewashed HPTLC silica gel 60 plates with the following settings: band length—8 mm, dosage speed—80 nL/s, application volume—between 1–8 µL, syringe installed—10 µL. The development was performed with a mixture of toluene—ethyl acetate, 2:3 (*v*/*v*) up to 80 mm after pre-conditioning with toluene, with a pump power of 40% for 150 s, followed by drying for 5 min. For the MMC, the mobile phase was toluene—ethyl acetate—methanol 2:1:1 (*v*/*v*/*v*), up to 80 mm, without preconditioning. The humidity was controlled at 0% during both elutions using a molecular sieve. The application and elution were performed using HPTLC PRO (CAMAG). The bioautograms were documented (TLC Visualizer 2, CAMAG, Muttenz, Switzerland) at fluorescence light detection (FLD) 254 nm and 366 nm, along with white light illumination. 

### 2.5. Planar Umu-C Bioassay 

The ON culture described above was subdivided by 1:7.5 dilution with LB medium and incubated at 37 °C and 150 rpm for 2 h. This bacterial culture of 70–80% of the initial OD_600_ was centrifuged (3000× *g*, 10 min) and re-suspended in LB medium to obtain a *Salmonella* suspension with an OD_600_ of 0.2. The bacteria suspension was sprayed onto the plate using the Chromajet DS20 (Biostep, Burkhardtsdorf, Germany) as follows: reagent quantity—4.01 mL, spray cycles–3, width—200 mm, length—100 mm. The plate was incubated in a dark plastic box with nearly 100% relative humidity for 3 h at 37 °C and dried for 4 min in a stream of cold air. For detection of the β-galactosidase activity, a phosphate buffer (10 mL) containing FDG (100 µL, 5 mg/mL in DMSO/H_2_O 1:1, *v*/*v*) was sprayed, as before. After 15 min incubation in a dark plastic box with 100% relative humidity at 37 °C, the fluorescence of the fluorescein was measured at 485/>500 nm (tungsten lamp, TLC Scanner 3, CAMAG, Muttenz, Switzerland)). The bioautograms were documented at a fluorescence light detection (FLD) of 254 nm and 366 nm, along with white light illumination.

### 2.6. Metabolic S9-Activation 

For the test condition in presence of metabolic activation, a mixture of bacteria suspension and S9-mix (0.5 mL, containing, according to the manufacturer, 1.916 mL buffer salts, 0.084 mL G-6-P, and 0.332 mL NADP, *i.e.*, 18% S9) was sprayed, as previously described. 

For the S9 optimization, different amounts of AFB1 from 10 to 800 pg/band, a methanol solvent control, and 4-NQO (500 pg/band) as a positive control were applied on three different plates. Therefore, three S9-mixtures containing *Salmonella* suspensions (OD = 0.2) were prepared by adding 0.1, 0.5, and 1 mL S9 (1%, 5% and 10% S9 respectively), to bacteria suspension to obtain 10 mL of the mixture, following the protocol as described. 

### 2.7. SOS Umu-C Assay Protocol 

The ON culture was prepared as described. The assay was performed according to the ISO guideline [20], as stated by Xenometrix, with minor modifications. Briefly, the bacterial culture of 70–80% of the initial OD_600_ was used for the assay. For each well of the 96-well plate (Thermo Fisher Scientific, Roskilde, Denmark), test substances, positive (4-NQO at 0.5 µg/mL in the absence, and 2-AA at 2 µg/mL in the presence, of metabolic activation) and negative controls (only bacteria suspension) were tested in biological triplicates. Bacteria culture was added to each well and mixed with the samples and controls. For the metabolic activation, a mixture of 30% S9-mix in bacteria culture was added to each well instead. The two plates were incubated at 37 °C and 150 rpm for 2 h. Then, bacteria were diluted 10 times in a new plate with fresh media. The absorbance at 600 nm was measured. The plates were incubated for another 2 h under the same conditions. Bacteria were again diluted ten times in a new plate, mixed with a mixture of B-Buffer/ONPG, and incubated for 30 min at 37 °C and 150 rpm. After adding a stop reagent into each well, the absorbance at 420 nm was measured to evaluate the conversion rate of β-galactosidase. 

### 2.8. Liquid Ames MPF Protocol 

The liquid Ames MPF method was applied, as recommended by the supplier [21]. Briefly, overnight grown *Salmonella* bacteria strains TA-98 for frameshift mutations and TA-100 for point mutations were exposed, in medium containing histidine in 24-well plates, to compounds at increasing concentrations in the presence or absence of metabolic activation at 37 °C for 90 min. Then, bacteria were diluted into a pH indicator (bromocresol purple) medium lacking histidine using 384-well plates and incubated at 37 °C for 48 h. The bromocresol purple turned yellow as the pH dropped due to the catabolic activity of revertant cells, which grew in the absence of histidine. The number of wells containing revertant colonies were counted and compared to the solvent control (DMSO). The cytotoxicity of the compounds tested in the AMES MPF test was estimated with the BacTiter-Glo^TM^ Microbial Cell Viability assay by adding a single reagent directly to the medium containing exposed bacteria, measuring the luminescence. The luminescent signal is proportional to the amount of the ATP present, which is directly proportional to the number of viable cells in the culture.

### 2.9. Data and Statistical Analysis 

The HPTLC biodensitograms were evaluated considering the peak height (visionCATS 3.0 software, CAMAG, Muttenz, Switzerland). Data were analyzed using the average of triplicates performed for each pure compound, considering the dose–response effect. Twenty peaks in the solvent control track were used as the mean blank. The results were expressed as induction ratio (*IR*) determined by the average of the normalized peaks. The *IR* was calculated according to Equation (1), where H_n_ is the peak height for the three compound replicates, and AVGb + Sd is the average peak height plus standard deviation of 3 blanks at a similar *hR*_F_ to the analyte.

(1) $$IR=(\left(\frac{H1}{\mathrm{AVGb}+\mathrm{Sd}}\right)+\left(\frac{H2}{\mathrm{AVGb}+\mathrm{Sd}}\right)+\left(\frac{H3}{\mathrm{AVGb}+\mathrm{Sd}}\right))/3$$

The data obtained were evaluated according to the guidelines of the International Conference on Harmonization [22]. The experiments were conducted in biological triplicates for each compound, in the presence and absence of metabolic activation. The repeatability was expressed as the relative standard deviation of peak height (%RSD) calculated using the ratio between the standard deviation and the average of the triplicates. The linearity was fitted with the first five doses of the dose–response curve, confirming the coefficient of determination for acceptable linear relationship (*r*^2^ ≥ 0.99).

For the liquid Umu-C assay, biological triplicates were performed. The relative units (RU) for each replicate were obtained at OD_600_ for the growth factor (*G*), and OD_420_ for the IR. Data were analyzed using the average of the triplicates performed for each compound and the corresponding standard deviation. The quality criteria to classify a sample as genotoxic with respect to the blank and negative controls is a *G* ≥ 0.5 and *IR* ≥ 1.5, as recommended by the supplier. 

For the liquid Ames MPF assay, biological triplicates were performed. Data were analyzed using the proprietary Xenometrix Calculation Sheet Version 3.23u 4/2017. Briefly, the mean number of positive wells (yellow) out of 48 wells per replicate and dose was compared with the number of spontaneous revertant wells obtained in the negative control samples. The fold increase (FI) above the baseline (mean of negative controls, *n* = 3, plus 1 standard deviation) was determined for each dose of a test chemical. Quality controls were applied for assay validity, considering concentrations with FI ≥ 2.0 (for TA100) and FI ≥ 3.0 (for TA98) as mutagenic concentrations. 

The limit of detection (LOD) and the limit of quantification (LOQ) of selected compounds were calculated to determine the lowest activity that can be detected with acceptable precision (LOD) and that can be quantitated with a degree of certainty (LOQ), according to the International Conference on Harmonization (ICH) [22] guidelines using the three- and ten-fold standard deviation of blank peak heights divided by the slope of the regression line. The lowest effective concentration (LEC) is the lowest concentration with an IR above the 2-fold threshold, according to supplier’s recommendation. To ensure comparability with the LECs of the Umu-C microtiter and AMES MPF bioassays at *IR* ≥ 1.5 and 2.0, respectively, the LEC of the HPTLC-SOS-Umu-C bioassay was set as the lowest concentration with an IR above 2.0. Data graphs were produced using GraphPad Prism 9.0.0 (GraphPad Software LLC, San Diego, CA, USA).

The limit of biological detection (LOBD) was applied, as previously reported [3,6]. This concept proposes the consideration of not only the above analytical detection capability, but also the contribution of the conditions applied for sample preparation and bioassay exposure, including the migration protocol, migrate concentration, and final exposure to the bioassays. This approach considers the concentration and dilution factors during sample preparation and the corresponding factor applied to the LEC. In the case of the Ames test, this corresponds to a concentration factor of 40× for 1 L of migrate concentrated 1000×, followed by a 25× dilution during cell treatment. To allow for direct comparisons, a maximum 1000× migrate concentration factor (1 L migrate concentrated 1000× to 1 mL directly loaded on the HPTLC plate), along with the measured LECs, was considered for HPTLC-SOS-Umu-C.

The target limit of biological detection (tLOBD) is defined as the LOBD required to comply with a predefined level of health risk (carcinogenicity). The tLOBD was estimated, considering the total daily dose calculated to produce an excess carcinogenic risk of 1 in 100,000 applied to 1 kg of packaged food for a 60 kg adult [3]. 

### 2.10. Selection of Reference Compounds 

The developed method was applied to 11 pure compounds to demonstrate the repeatability and the validity of the protocol. To avoid false positive results, the selection of the test substances was based on the recommendations regarding chemicals that would be appropriate for evaluating the sensitivity and specificity of the genotoxicity test [19]. Based on those recommendations, for the selection of compounds for the HPTLC coupled with the Umu-C assay, the following criteria were applied: applied DNA-damage with diverse mechanisms of actions, including direct DNA-binding, indirect DNA-damage, and negative controls (Table 1). 

## 3. Results

### 3.1. Incorporation of a Metabolic Activation Step 

**Figure 1:**
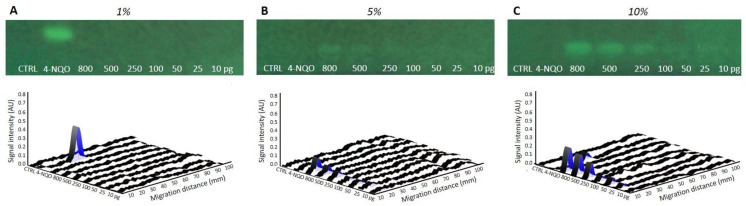
Bioautogram at FLD 254 nm showing the region of interest for optimization of the metabolic activation varying percentage (%) of mixtures of the S9 fraction, with co-factors, at (**A**) 1%, (**B**) 5%, and (**C**) 10%. Biodensitograms at 485/>500 nm of AFB1 (10−800 pg/band), along with the positive control 4-NQO (500 pg/band) and methanol solvent control (CTRL, 10 µL/band).

The incorporation of a metabolic activation step in the latest HPTLC-genotoxicity bioassay [8] was undertaken by spraying a mixture of bacteria and rat liver S9-fraction, containing P450 enzymes and required cofactors, directly onto the chromatogram. The ratio between the S9 and the bacteria was investigated to obtain optimal genotoxic signals. Biological triplicates of S9-*Salmonella* preparations, with fixed amounts of bacteria but increasing levels of S9-mix, corresponding to final concentrations of 1%, 5%, and 10%, were evaluated on plates loaded with AFB1 (requiring S9-activation) and 4-NQO (inactivated by S9). With AFB1, no response was visible on the bioautograms and densitograms of the plates sprayed with 1% S9 mixture (Figure 1A). However, clear dose-dependent signals were observed at higher S9 concentrations, with the best response obtained at 10%. With the standard control 4-NQO, a signal was found on the plate sprayed with 1% S9-mix, but not at higher S9-concentrations, indicating a metabolic inactivation at the dose tested (Figure 1B,C). 

**Figure 2:**
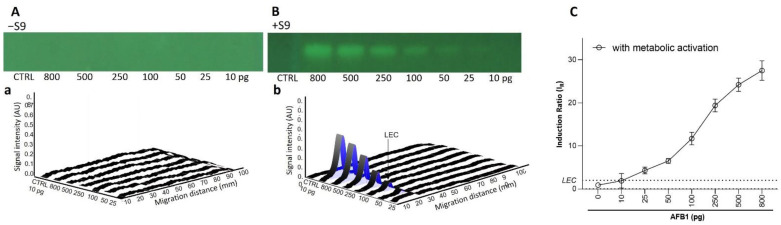
Dose–response effect for AFB1. Bioautograms at FLD 254 nm (region of interest shown) in absence (−S9) (**A**) or presence (+S9) (**B**) of metabolic activation, with respective biodensitograms at 485/>500 nm (a and b). Dose–response curves with LEC (black dotted line at IR 2.0) (**C**).

**Figure 3:**
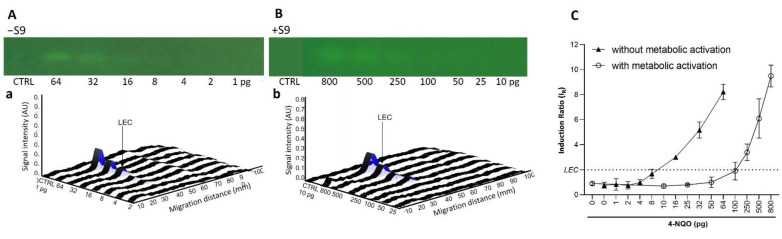
Dose–response effect for 4-NQO. Bioautograms at FLD 254 nm (region of interest shown) in absence (−S9) (**A**) or presence (+S9) (**B**) of metabolic activation, with respective biodensitograms at 485/>500 nm (a and b). Dose–response curves with LEC (black dotted line at IR 2.0) (**C**).

To confirm these results and to assess the repeatability of the test, dose-response curves in the absence (−S9) and presence (+S9, 10%) of metabolic activation were generated in biological triplicates. As expected for the AFB1 (10−800 pg/band; Figure 2), a dose-dependent genotoxic response was only observed in the presence of metabolic activation. A genotoxic signal for 4-NQO (1–64 pg/band without S9, 10–800 pg/band; Figure 3) was detected in both the absence and presence of the S9-mix. However, for this chemical, the dose–response curves obtained in the presence of metabolic activation were clearly shifted towards the right, confirming the S9-dependent inactivation. With both substances, the results were highly reproducible. The LECs in the presence of metabolic activation for AFB1 and 4-NQO were determined at 25 (0.08 pmol) and 250 pg/band (1.3 pmol), respectively. The LEC of 4-NQO in the absence of metabolic activation was determined at 16 pg/band (0.08 pmol). 

**Figure 4:**
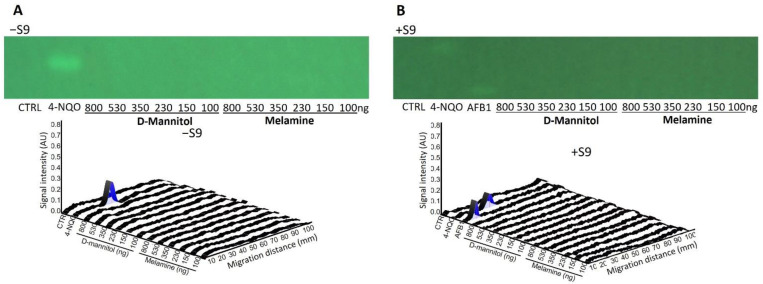
Bioautograms at FLD 254 nm (region of interest shown) and biodensitograms at 485/>500 nm showing the negative controls D-mannitol, melamine (100−800 ng/band each), and with the positive control 4-NQO (500 pg/band) (**A**) in absence (−S9) and (**B**) in presence (+S9) of metabolic activation.

D-mannitol and melamine (100−800 ng/band) were selected as negative controls to demonstrate the specificity of the test. As expected, no genotoxic response was observed in the corresponding bioautograms and densitograms for both substances (Figure 4). The expected responses were observed with AFB1 and 4-NQO, at 500 and 800 pg/band respectively, confirming the validity of the test. 

### 3.2. Performance of the HPTLC-SOS-Umu-C Assay

**Figure 5:**
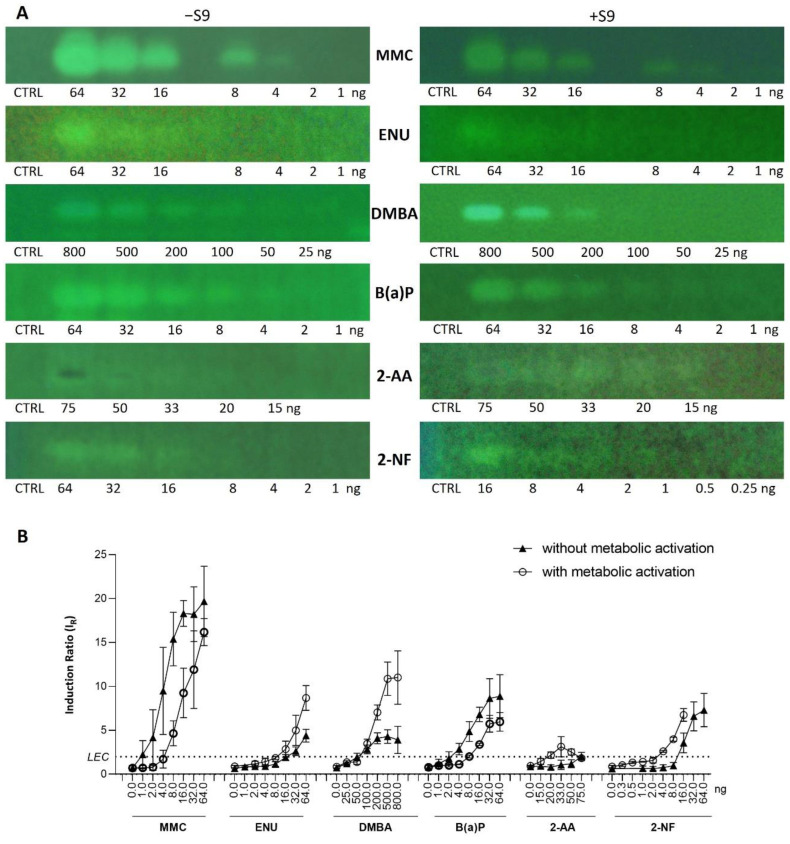
Dose–response study extended to 6 compounds (Table 1) in absence (−S9) and presence (+S9) of metabolic activation, along with methanol solvent control (CTRL). (**A**) Bioautograms at FLD 254 nm (region of interest shown) and (**B**) dose–response curves with LECs (black dotted line at IR 2.0).

Seven additional compounds (Table 1) were selected to further demonstrate the predictive value of the full p-Umu-C test protocol, in the presence and absence of the S9-mix. The selection was based on reported ECVAM recommendations for validation of new or modified genotoxicity tests [19], including chemicals causing DNA damage through diverse mechanisms of actions. The dose–response effects were studied in the presence and absence of metabolic activation and in triplicates (Figure 5A; one representative bioautogram replicate is shown). The applied amount was adjusted according to responses obtained in preliminary experiments (1−64 ng/band for MMC, ENU, and B(a)P; 25–800 ng/band for DMBA; 15–75 ng/band for 2-AA; 1–64 and 0.25–16 ng/band for 2-NF). The obtained fluorescence signals were normalized to the solvent control background signal to calculate the induction ratio and LEC (Figure 5B). A clear dose–response effect was observed for six test compounds in the presence and absence of metabolic activation. The genotoxic response of 2-AA was weak; however, an inhibitory dark signal was noticed on the bioautogram, possibly reflecting bacterial toxicity. B(a)P and DMBA both showed positive signals in the absence and presence of metabolic activation. 

**Figure 6:**
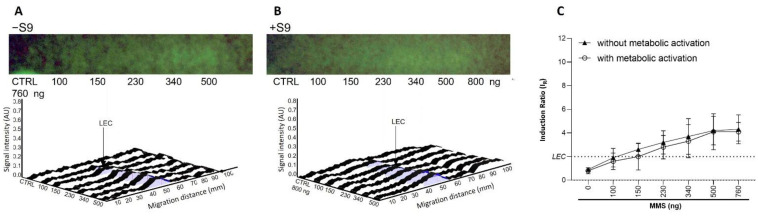
Dose–response study for MMS (100–760 ng/band) (**A**) in absence (−S9) and (**B**) in presence (+S9) of metabolic activation. Bioautograms at FLD 254 nm (region of interest shown), biodensitograms at 485/>500 nm and (**C**) dose–responses curves with LEC (black dotted line at IR 2.0).

Variable band diffusion was observed, possibly complicating the data analysis. This is illustrated by the data obtained with MMS in both the presence and absence of metabolic activation (Figure 6). Despite the impaired band visualization on the bioautograms at 254 nm, the dose–response curves were still usable in the respective densitograms of the fluorescence measurement at 485/>500 nm. Measuring the peak area instead of the height did not provide any improvement, significantly increased variability. 

The data obtained were evaluated according to the guidelines of the International Conference on Harmonization [22]. The experiments were conducted in biological triplicates for each compound, in the presence and absence of metabolic activation. The data were used to establish LODs, LOQs, and method repeatability (Table 2). The repeatability was expressed as the relative standard deviation of peak height (*%RSD*) calculated using the ratio between the standard deviation and the average of the triplicates. The linearity was fitted with the first five doses of the dose–response curve, confirming the acceptable linear relationship (*r*^2^ ≥ 0.99), with two exceptions (*r*^2^ ≥ 0.96 for 2-AA and MMS) (data not shown). In the presence of metabolic activation, the *%RSD* of AFB1 was 12%, and of DMBA, ENU, 2-NF, 2-AA, and B(a)P, up to 20%. The *%RSD* was 23% for 4-NQO, and due to diffusion, 38% for MMS. In the absence of metabolic activation (AFB1 excluded), the *%RSD* was 24%, 20%, and 18% for 4-NQO, DMBA, and ENU, respectively, and approximately 30% for the other compounds. The acceptance criteria established by the ICH guidelines is *%RSD* ≤ 20%, suggesting an overall acceptable performance of the HPTLC SOS Umu-C assay. However, some exceptions were observed for MMC and MMS, which are more related to the analytes’ features and not necessarily to the performance of the assay. 

### 3.3. Performance Comparison with Standard Multi-Well Liquid Format Assays 

LECs for 4-NQO and AFB1 obtained in the AMES MPF and Umu-C microtiter plate assays (expressed in ng/well) were compared to those generated using the HPTLC−S9-Umu-C bioassay (Table 3). Data show consistently lower LECs for the HPTLC method, suggesting a high potential for this new methodology to significantly reduce the limit of biological detection of genotoxic substances in complex mixtures, such as packaging migrates. 

To estimate the potential improvement in detecting low levels of genotoxic substances in FCM migrates requires considering not only the intrinsic analytical capability of the test (reflected in the LECs), but also the sample preparation protocol, including migrate concentration and dilution prior to bioassay exposure. This allows for the calculation of the limits of biological detection (cLOBDs) in migrate samples [3,7]. To do this, the approach previously developed to assess the standard Ames test was used [3,7]. Data on the chemicals for which all necessary information was available are provided in Table 4. Ratios between calculated cLOBDs for Ames (cLOBDs_Ames_) and HPTLC (cLOBDs_HPTLC_) assays highlight that cLOBDs_HPTLC_ were, in general, orders of magnitude lower than those anticipated according to the use of the standard Ames test. The cLOBDs_HPTLC_ were also compared with target LOBDs (tLOBDs) established to limit an excess carcinogenic risk of 1 in 10^5^ [3]. Ratios between tLOBDs and cLOBDs_HPTLC_ range from 1.2 (MMC) to 260 (MMS), indicating that in general, the cLOBDs_HPTLC_ are equal to or lower than what would be required from a safety perspective. Interestingly, all chemicals were detected at a concentration corresponding to the threshold of toxicological concern for substances presenting structural alerts for genotoxicity (150 ng/person, converted in 150 ng per band [3]).

## 4. Discussion

In vitro bioassays aimed at determining genotoxic/mutagenic potential are standard tests required for chemical risk assessment [25]. They have been designed and successfully applied for evaluating pure substances. However, their suitability for characterizing complex chemical mixtures containing unidentified components is being challenged [3,4,5,6,7]. Indeed, an adequate test should possess the capability to detect genotoxicants/mutagens at levels low enough to be compatible with negligible carcinogenic risk [3]. Currently, there is no test available exhibiting such a high level of performance. This lack was recently highlighted in the field of packaging safety [3,5]. Although the Ames assay is recommended as the best possible choice to test packaging migrates, it is also recognized that it suffers from significant weaknesses with respect to limits of biological detection [3]. Research efforts are therefore necessary to address this important limitation. In this context, the development of alternative methodologies using HPTLC coupled with genotoxicity assays, such as the SOS-Umu-C assay, has been encouraging [7], providing potentially improved detection limits compared to standard methods. However, until now, the reported protocols have been applied mainly to 4-NQO in the absence of metabolic activation. Thus, there is a need to confirm the promises of the p-Umu-C test through the detection of more chemicals acting through different mechanisms of action and to incorporate metabolic activation. 

The current study expands the number of chemicals tested in the p-Umu-C test. The data provides new evidence confirming the capability of this test to detect low levels of DNA-damaging substances. The main key contribution of the study is the development and implementation of metabolic activation into the previously published protocol [7]. AFB1 was used as a prototypical reference compound requiring bioactivation. Standard post-mitochondrial S9-fraction was employed as a source of xenobiotic metabolizing enzymes. The generated data demonstrated the efficacy of the developed S9-mediated bioactivation protocol, with reduced assay time as compared to other classical methods (~6 h/without considering the ON culture). Altogether, these data provide assurance that pro-mutagens requiring bioactivation to express their genotoxic potential can be detected with the p-Umu-C assay. With some other substances, such as 4-NQO and MMC, a shift in the genotoxic activity dose–responses to the right was observed, suggesting a possible S9-mediated inactivation. The establishment and incorporation of an S9-protocol in the p-Umu-C approach may be considered as a breakthrough step for the acceptability of this method as a credible genotoxicity test.

Among the reference genotoxic chemicals used, 4 were polycyclic aromatic hydrocarbons (PAHs, *i.e.*, 2-NF, 2-AA, DMBA, and B(a)P). In agreement with published data, 2-NF induced genotoxic activity in both the presence and absence of S9 [24]. However, the 3 others, which are documented to require bioactivation to exert their genotoxic potential, were also active in the absence of S9 treatment. The role of the metabolic activation of PAHs has been extensively studied. For example, it is well known that in biological systems, B(a)P must be converted to oxygenated metabolites to exert its mutagenic activity. Cytochrome P-450 oxidase-mediated activation is involved in these reactions, forming the DNA-reactive metabolites, *e.g.*, the 7,8-diol-9,10-epoxide [26]. However, evidence is available that the non-enzymatic transformation of PAHs such as B(a)P may also occur, for example, through photo-activation by irradiation with UV light [27,28]. In this type of reaction, genotoxic derivatives are formed, such as quinones, which can either directly bind to DNA, or induce damages through the production of H_2_O_2_ by redox cycling [29,30,31]. The physicochemical conditions present in the separation step of the p-Umu-C assay or the recording of UV images after the separation may be favorable to the non-enzymatic oxidation of PAHs and therefore, constitute a plausible explanation for the induction of genotoxic activity observed with PAHs in the absence of S9. This deserves further attention and investigations.

The main promise of HPTLC coupled with genotoxicity assays is its high potential for application to complex mixtures containing active substances at very low levels. Indeed, because of their possible high potency, low levels of genotoxic chemicals may still be of significant safety relevance and require identification. Currently, no satisfactory test is available for this purpose [3,4,5,6,7]. The HPTLC-bioassay approach was chosen to specifically address this issue. LECs for AFB1 and 4-NQO obtained in the p-Umu-C assay were found to be much lower than those observed in liquid formats of the SOS Umu-C and Ames tests, suggesting a real potential for this approach to achieve the detection of levels of genotoxic chemicals that are compatible with safety. This was confirmed with the theoretical LOBDs in migrates calculated for 7 of the tested chemicals. These were orders of magnitude lower than those established for the standard Ames test. Importantly, the calculated LOBDs obtained with the p-Umu-C assay were in line with levels associated with negligible carcinogenic risk, as calculated based either on TD50s, or on the threshold of toxicological concern (TTC) for substances bearing alerts regarding genotoxicity [3]. Although the application of the method to case studies involving packaging matrices is still required, the data generated up to this point indicate that the p-Umu-C bioassay may contribute toward filling the actual gaps and satisfying the other limitations of microplate-based in vitro testing for decision-making and prioritization purposes.

It must be acknowledged that the p-Umu-C test cannot readily replace the Ames test, since it does not specifically detect mutagens. This can be seen as a limitation regarding packaging safety assessment [3]. However, the active bands on HPTLC-plates can be recovered and then either identified chemically by high resolution mass spectrometry or/and directly tested in a suitable Ames assay format. This should provide highly relevant information regarding mutagenic potentials of the chemicals present in the band.

In conclusion, the p-Umu-C incorporated with the S9 metabolic activation condition has the potential to become the most suitable approach to identify genotoxic/mutagenic substances in complex mixtures, such as packaging migrates, or food and environmental samples. It appears to be the tool of choice to support the application of the TTC Cramer class III threshold to prioritize unidentified substances in migrates, as recently proposed.

## Figures and Tables

**Table 1:** Selected reference compounds with different mechanisms of action.

Compounds	CAS No.	Chemical Structure	Comments		Classification
4-Nitroquinoline-*N*-oxide (4-NQO)	56-57-5	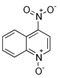	Alkylating agent, forms DNA adducts	In-vivo genotoxins which should be detected with positive response in in-vitro genotoxicity tests	
Aflatoxin B1 (AFB1)	1162-65-8	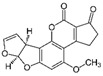	Requires metabolic activation; forms various adducts		
Mitomycin C (MMC)	50-07-7	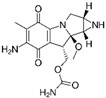	Inducing DNA–DNA crosslinks and oxidative damage; alkylating activity		
Methyl methanesulfonate (MMS)	66-27-3	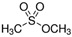	Strong clastogen		
*N*-ethyl-nitrourea (ENU)	759-73-9	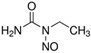	Strong gene mutation		
7,12-Dimethylbenzanthracene (DMBA)	57-97-6	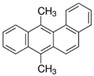	Requires metabolic activation. Forms bulky adducts		
Benzo(a)pyrene (B(a)P)	50-32-8	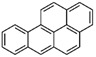	Requires metabolic activation; forms bulky adducts		
2-Aminoanthracene (2-AA)	613-13-8	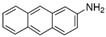	Requires metabolic activation; forms bulky adducts [23]		
2-Nitrofluorene (2-NF)	607-57-8	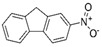	Forms DNA adducts [24]		
D-mannitol (D-man)	69-65-8	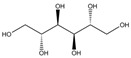	*n*/*a*	Non-DNA reactive chemicals that should give negative results in in-vitro genotoxicity tests	
Melamine	108-78-1	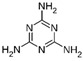	*n*/*a*		

**Table 2:** Performance data for 9 reference compounds tested in the HPTLC-SOS-Umu-C assay in the presence and absence of metabolic activation.

Substances	Without Metabolic Activation						With Metabolic Activation					
ng/band	LEC	LOD	LOQ	Linearity Dose Range	*r* ^2^	% RSD	LEC	LOD	LOQ	Linearity Dose Range	*r* ^2^	% RSD
4-NQO	0.016	0.006	0.021	0.001–0.016	0.997	17	0.25	0.056	0.186	0.01–0.25	0.996	23
AFB1	−	−	−	*n*/*a*	*n*/*a*	−	0.025	0.006	0.022	0.01–0.25	0.994	17
MMC	1.0	0.40	1.32	1–16	0.991	33	4	1.19	3.95	1–16	0.997	35
MMS	150	119.74	399.14	100–500	0.966	29	230	116.49	388.30	100–500	0.986	33
ENU	16	8.70	28.99	1–16	0.992	18	16	6.97	23.24	1–16	0.992	19
DMBA	50	37.48	124.94	25–500	0.992	20	100	33.35	111.15	25–500	0.995	16
B(a)P	4	1.42	4.72	1–16	0.993	28	8	3.37	11.24	1–16	0.993	19
2-AA	75	37.23	124.10	15–75	0.961	32	20	14.73	49.12	15–75	0.986	17
2-NF	16	5.66	18.87	1–16	0.995	32	4	2.14	7.13	0.25–4	0.994	9

(−): negative in absence of metabolic activation; LEC: lowest effective concentration; LOD: limit of detection; LOQ: limit of quantification; (% *RSD*): relative standard deviation; *r*^2^: coefficient of determination.

**Table 3:** Comparison of the LECs of the p-Umu-C, SOS Umu-C and AMES-MPF (TA98 and TA100) assays. For liquid test formats, data are expressed in ng/well. For p-UmuC, data are expressed in ng/band.

Condition	LEC (ng) without Metabolic Activation				LEC (ng) with Metabolic Activation			
Assays	HPTLC Umu-C	Liquid Umu-C	AMES-MPF		HPTLC Umu-C	Liquid Umu-C	AMES-MPF	
Strains			TA98	TA100			TA98	TA100
4-NQO	0.016	25	15.5	6.25	0.25	50	395 *	79 *
AFB1	−	−	−	−	0.025	47.8	0.06	0.24

(−): negative in absence of metabolic activation; * data kindly provided by Xenometrix.

**Table 4:** Comparison of the calculated LOBDs for theHPTLC−S9-Umu-C versus the standard Ames bioassays for selected genotoxic substances.

Genotoxic Substances	cLOBD_HPTLC_ (ng/L)	* cLOBD_Ames_ (ng/L)	cLOBD_Ames_/cLOBD_HPTLC_	* tLOBD (ng/L)	tLOBD/cLOBD_HPTLC_
AFB1	0.025	35	1400	3.9	156
MMC	1	40	40	1.2	1.2
MMS	150	175	1.167	39,000	260
ENU	16	300	18.750	1000	63
DMBA	50	200,000	4000	100	2
B(a)P	20	5000	250	1100	55
2-NF	20	5000	250	340	17

* From Schilter et al. [3]; cLOBD: (calculated LOBD); tLOBD: (targeted LOBD).

## Data Availability

Not applicable.
